# Death of Professor Dugas

**Published:** 1884-11-20

**Authors:** 


### *DEATH OF PROFESSOR DUGAS.*

An obituary notice of this distinguished son of Georgia, prepared when going to press with our last issue, was, by some oversight, left out. He died at his home in Augusta on the 10th of October, 1884, aged 78 years. For more than fifty years he was a Professor in the Medical College of Georgia, at Augusta, serving during the time in the different chairs of Anatomy, Physiology, and Practice. He was a man of original and progressive mind, a profound student, and an able lecturer.

He was a gentleman of high moral and intellectual characteristics, and always maintained a high and honorable position, not only in the Profession but as a man and a citizen.

